# Human parvovirus PARV4 DNA in tissues from adult individuals: a comparison with human parvovirus B19 (B19V)

**DOI:** 10.1186/1743-422X-7-272

**Published:** 2010-10-15

**Authors:** Fabiana Corcioli, Krystyna Zakrzewska, Rosa Fanci, Vincenzo De Giorgi, Massimo Innocenti, Matteo Rotellini, Simonetta Di Lollo, Alberta Azzi

**Affiliations:** 1 Department of Public Health, University of Firenze, Viale Morgagni 48, Firenze, Italy; 2 Department of Hematology, University of Firenze, Viale Morgagni 85, Firenze, Italy; 3 Department of Dermatological Sciences, University of Firenze, Viale Morgagni 85, Firenze, Italy; 4 Department of Orthopedics, Traumatology, Plastic surgery and Rehabilitation, University of Firenze, Viale Morgagni 85, Firenze, Italy; 5 Department of Human Pathology, University of Firenze, Viale Morgagni 85, Firenze, Italy

## Abstract

**Background:**

PARV4 is a new member of the Parvoviridae family not closely related to any of the known human parvoviruses. Viremia seems to be a hallmark of PARV4 infection and viral DNA persistence has been demonstrated in a few tissues. Till now, PARV4 has not been associated with any disease and its prevalence in human population has not been clearly established. This study was aimed to assess the tissue distribution and the ability to persist of PARV4 in comparison to parvovirus B19 (B19V).

**Results:**

PARV4 and B19V DNA detection was carried out in various tissues of individuals without suspect of acute viral infection, by a real time PCR and a nested PCR, targeting the ORF2 and the ORF1 respectively. Low amount of PARV4 DNA was found frequently (>40%) in heart and liver of adults individuals, less frequently in lungs and kidneys (23,5 and 18% respectively) and was rare in bone marrow, skin and synovium samples (5,5%, 4% and 5%, respectively). By comparison, B19V DNA sequences were present in the same tissues with a higher frequency (significantly higher in myocardium, skin and bone marrow) except than in liver where the frequency was the same of PARV4 DNA and in plasma samples where B19V frequency was significantly lower than that of PARV4

**Conclusions:**

The particular tropism of PARV4 for liver and heart, here emerged, suggests to focus further studies on these tissues as possible target for viral replication and on the possible role of PARV4 infection in liver and heart diseases. Neither bone marrow nor kidney seem to be a common target of viral replication.

## Background

PARV4 is a member of the Parvoviridae family discovered in 2005 in plasma from an intravenous drug user, with symptoms consistent with acute HIV infection, but confirmed to be HIV RNA negative [1]. It seemed to be not closely related to any of the known human or animal parvoviruses until 2008 when novel porcine and bovine parvoviruses highly similar to PARV4 were identified so that it was proposed the inclusion of PARV4 in a new genus Hokovirus [2]. Three genotypes of PARV4 have been identified at present [3,4]. Till now, it has not been associated with any disease and its spread in the human population has not been clearly assessed. In fact, the serological assays for anti-PARV4 antibody detection, only recently developed, till now have been applied on small groups of individuals only [5]. Viremia seems to be a hallmark of PARV4 infection. PARV4 was found in 5% of plasma pools at levels suggesting the existence of donations with high viral load [6], in 16% of clotting factor VIII concentrates [7], in 2% of plasma samples from healthy donors [8] and in 7,95% of serum samples from intravenous drug users in Thailand [9]. Several data indicate that the transmission of the virus predominantly occurs by parenteral route [5,10] whereas the respiratory transmission seems to be unlikely [11]. As regards tissue distribution, PARV4 DNA was demonstrated frequently in bone marrow and lymphoid tissue from HIV infected individuals [12] and also in liver of non HIV-infected adults [13]. One study reports that PARV4 DNA can be detected, with a low frequency, in healthy and pathological skin samples [14].

It is known that B19V frequently persist in solid tissues in asymptomatic individuals and, less frequently, also in bone marrow [15].

The aim of this study was to assess whether PARV4 is able to persist in different tissues, like B19V, or if it shows a particular tissue tropism.

## Results

With the aim to assess the tissue distribution and the ability to persist of PARV4 in comparison to B19V, we searched for DNA sequences of both parvoviruses in various tissues of individuals without suspect of acute viral infection. Table 1 shows the results of PARV4 DNA detection in different tissues, in comparison with B19 DNA detection. The frequency of PARV4 DNA finding was low in skin, synovium and bone marrow (4-5,5%) whereas in myocardium samples PARV4 sequences were shown in 17 out of 35 (49%), a frequency significantly higher than in the other tissues examined but significantly lower than that of B19V. PARV4 DNA was found in 8 out of 39 plasma specimens (20,5%) while B19V DNA was demonstrated in one of these specimens only (2,5%). It is noteworthy that these 39 specimens were from 21 immunodepressed hematological patients and 18 immunocompetent individuals and the frequency of viremia was 33% (7 positives out of 21) in the former group and 5,5% (1 positive out of 18) in the second group (however, this difference is not statistically significant). The viral load in these sera was low: below 3,8 × 10^3^copies/ml.

**Table 1:** Detection of PARV4 DNA in tissues and plasma in comparison with B19 DNA in the same specimens*

Samples	N°	PARV4 DNA positive (%)	B19V DNA positive (%)
Bone marrow°	26	2 (5,5)	9 (25)

Skin°	25	1 (4)	18 (72)

Sinovyum	21	1 (5)	6 (28,5)

Myocardium°	35	17 (49)	26 (74)

Plasma°	39	8 (20,5)	1 (2,5)

*part of the results of B19 DNA detection have been previously reported (12)

°difference between percentages of PARV4 and B19V DNA statistically significant

From 17 out the 35 individuals dead for various causes, without known viral myocarditis or dilated cardiomyopathy, lungs, liver, kidney and blood specimens could be examined, in addition to the myocardium, with the aim to verify the distribution of PARV4 and B19V DNA in the different organs of the same individual. In none of these cases viral DNA was detectable in the blood while PARV4 as well as B19V DNA were found frequently in the different organs analyzed (Table 2). PARV4 DNA was present more frequently in the liver and in the myocardium (41%) than in lungs and kidneys (23,5% and 18% respectively). The frequency of PARV4 DNA in the liver was similar to that of B19V DNA in this tissue and lower in the others. In one individual PARV4 DNA and in 4 individuals B19V DNA were demonstrated in myocardium samples only. In one individual PARV4 DNA and in 4 individuals B19V DNA were present in all the tissues examined. In two cases PARV4 DNA sequences were not demonstrable in the myocardium and were detectable in other tissues, whereas B19V infected individuals had always DNA sequences in the myocardium.

**Table 2:** Detection of PARV4 and B19V DNA in different tissues from 17 individuals without known viral myocarditis or dilated cardiomyopathy

	myocardium		lung		liver		kidney	
**Patient**	**PARV4**	**B19V**	**PARV4**	**B19V**	**PARV4**	**B19V**	**PARV4**	**B19V**

1	-	+	-	-	-	-	-	-
2	-	-	-	-	-	-	-	-
3	+	+	-	+	-	-	-	+
4	-	+	-	-	-	-	-	-
5	-	+	-	+	-	-	-	+
6	+	+	-	+	-	-	+	-
7	+	+	+	+	+	+	-	+
8	+	+	-	-	+	-	-	-
9	-	-	-	-	+	-	+	-
10	+	+	+	+	+	+	-	+
11	-	+	+	+	+	+	-	+
12	+	+	-	+	+	+	-	+
13	-	+	-	-	-	-	-	-
14	-	+	-	-	-	+	-	-
15	+	-	+	-	+	-	+	-
16	-	+	-	-	-	+	-	+
17	-	+	-	-	-	+	-	+

Tot*	7 (41)	14 (82)	4 (23)	7 (41)	7 (41)	7 (41)	3 (18)	8 (47)

* number of positives (%)

The concentration of PARV4 DNA in all the positive tissues was generally low, below or near the level of quantization by the real time PCR here employed, corresponding to ≤ 380 copies/10^6^cells.

As previously reported, the copy number of B19V DNA varied from below the level of quantization by the real time PCR to 7 × 10^4 ^copies per 10^6 ^cells, except for two myocardium samples where about 10^6 ^copies per 10^6 ^cells were found (15).

PARV4 sequence analysis, aimed to establish the genotype of PARV4 present in the myocardium samples, could be performed on 10 isolates only, out of 17 positive samples, because of the low level of viral DNA. All were included in the genotype 2 (PARV5) after comparison with consensus sequences of the three genotypes (GenBank accession numbers HQ245657-HQ245666). As regards B19V, either genotype 1 or genotype 2 were present, with a slightly higher frequency of the 2.

PARV4 DNA was undetectable in the urine specimens of the bone marrow transplantation patients.

## Discussion and Conclusions

In this study low amount of PARV4 DNA was found frequently (>40%) in heart and liver of adults individuals, less frequently in lungs and kidneys (23,5 and 18% respectively) and was rare in bone marrow, skin and synovium samples (5,5%, 4% and 5%, respectively). By comparison, B19V DNA was present in the same tissues with a higher frequency (significantly higher in myocardium, skin and bone marrow) except than in liver where its frequency was the same of PARV4 DNA and in plasma samples where B19V frequency was significantly lower than that of PARV4. Some of these results are fairly in agreement with the literature where the frequency of PARV4 DNA in bone marrow varies from 0% (0/8) in HIV uninfected individuals (like the patients enrolled in this study), to 46% and 65% in HIV infected patients [12,16] and is 7,7% in the skin of HIV-negative individuals [14]. The frequency of PARV4 DNA in the liver reported in literature [13] was lower (15%) in comparison with 41% in this study. It is to note that the study of Schneider [13] and the present study are very different as regards the number and the source of the specimens analyzed as well as the methods used for PARV4 DNA detection; in fact in the former study 87 liver specimens, obtained mainly in occasion of liver transplantation for various causes, were analyzed by four PCRs. The presence of PARV4 DNA in myocardium samples is reported here for the first time and with a high frequency. The viral load was low also in this tissue and 10 isolates out of the 17 positive myocardium samples that could be analyzed by sequencing showed to be genotype 2 (PARV5), a result that could be related with the age of these patients, all born before 1956 [12], in agreement also with B19V genotype distribution, as previously discussed [15].

Low level PARV4 viremia, which seems characteristic of persistent more than of acute infection, was more common in hematological immunodepressed patients (33%), in comparison with 5.5% in immunocompetent individuals. This result, if confirmed on a larger number of patients, could indicate that reactivation or persistence of PARV4 may occur under immunodepression conditions and manifest as viremia more frequently than B19V. In fact, B19V viremia was not reported in this group of hematological immunodepressed patients. Considering that PARV4 DNA was found in bone marrow from 2 out of 36 hematological patients only (5,5%) whereas viremia occurred in 7 out of 21 (33%) patients of the same group, these results could suggest that it is unlikely that the site of latency/persistence and reactivation of this virus is the bone marrow. In fact, the two bone marrow positive patients were also PARV4 DNA plasma positive, but at least for the other 5 plasma positive samples another source of viremia has to be hypothesized. On this point, the failure to detect urinary PARV4 shedding in the small group of bone marrow transplantation patients enrolled in this study seems indicate that viral reactivation in the kidney do not occur commonly. In addition, the failure to detect PARV4 DNA in BAL [14] seems indicate that lung is not a site of viral active infection, despite the presence of PARV4 sequences in 23% of individuals, as our results show. Further studies are necessary to detect the tissue target of PARV4 infection and the results here reported could suggest to focus in particular on the liver.

If more than 40% of individuals may have viral DNA sequences in liver or myocardium the parenteral route may not be the only way of PARV4 transmission. However, in agreement with the absence of respiratory transmission, it seems likely that PARV4 infection is less frequently transmitted than B19V infection and then it is less frequently detectable in the host tissues. When PARV4 infection occurs, the virus spreads to various organs where it can persist, more or less frequently. If confirmed, the observation that the frequency of PARV4 DNA persistence in the liver is of the same order than that of B19 DNA, while in all the other tissues here considered is lower, could indicate that the liver is a main target of PARV4 infection. Its particular tropism for myocardium and liver, here reported, suggests to further study the possible role of PARV4 infection in heart and liver diseases, considering also that PARV4 infection has been frequently detected in HCV infected patients. It is to note that also B19V is frequently detectable in liver and myocardial tissue both in acute and persistent B19V infection and its importance in liver and heart diseases still remains unclear [17-21].

## Methods

### Clinical samples

Tissue biopsies were collected in 2006, from 72 Italian adult patients, non HIV-infected, after informed consent: twenty six bone marrow samples from patients (mean age 60; range: 22-82) with B19-unrelated hematological disorders referred to the Department of Hematology, submitted to different chemotherapeutic regimens; twenty one synovial tissue samples from healthy individuals (mean age:70, range:36-82) who underwent surgery for arthrosis or joint trauma, referred to the Department of Orthopedics and Traumatology; twenty five skin biopsies from patients (mean age: 67, range 33-93) with B19-unrelated dermatological lesions, referred to the Department of Dermatological Sciences. All the patients were immunocompetent except those with hematological disorders. From 39 out of the 72 patients a serum sample was collected at the same time of the biopsy. In addition, from 2006 to 2010, myocardium autoptic samples were obtained from 35 Italian individuals, non HIV-infected (mean:70, range: 53-84) without known viral myocarditis or dilated cardiomyopathy. From 17 out of 35 cadavers autoptic samples from lungs, liver, kidney and blood were also available. In addition, 93 urine specimens from 29 bone marrow transplantation recipients, at different times after infusion were obtained for PARV4 DNA detection.

### PARV4 DNA detection and sequencing

After DNA extraction using the High Pure PCR Template Preparation Kit (Roche, Basel, Switzerland) PARV4 DNA detection was performed by two PCR able to amplify sequences of the three genotypes. A real time PCR using the primers PVORF2F and PVORF2R reported by Fryer (8) and Syber Green as intercalating dye, was developed for virus detection and viral load assessment. One μl (25 pmol) of each primer and 5 μl of extracted DNA were added to 18 μl of Quantitec SYBR Green PCR Master Mix (Qiagen, Hilden, Germany). A first step of 10 min at 94°C, to activate the Taq polymerase, was followed by 40 cycles of 94°C for 30 s, 62°C for 30 s, 72°C for 60 s, with a reading step at 79°C for 15 s. The reaction was performed using the Rotor-Gene 3000 real time apparatus (Corbett Research, Sidney, Australia).

Melting curve analysis was performed to control the specificity of the PCR products.

To construct the calibration curve, serial dilutions of the target sequence, 268 bp long, cloned in the p-GEM-T vector system (Promega, Milano, Italy) were used. The range of linearity of the quantitative reaction was 1,96 × 10^1^-1,96 × 10^6 ^copies/ml. A real time PCR for the β-globin gene was performed to calculate the number of cells of the tissues extracted, as already described [15]. About 50.000 cells per reaction were analyzed. In addition, a nested PCR described in literature [8], with primers targeting a conserved region of ORF1 of the three known PARV4 genotypes was used to confirm the results of the previous assay.

The sensitivity of these two assays was similar, both being able to detect 2 copies of its target/μl in 100% of cases.

A sample was considered PARV4 DNA positive if positive by both reactions.

Amplicons obtained by the real time PCR (ORF2) and by the nested PCR (ORF 1) were sequenced using the primers PVORF2F and PVORF2R and the inner primers, respectively. Sequencing was carried out by the dideoxynucleotide chain termination method on an ABI Prism 377 automatic sequencer (Applied Biosystems), using the ABI PRISM Dye Terminator cycle sequencing Ready Reaction kit.

B19 DNA detection and genotyping was performed as previously described [15].

### Statistical analysis

The frequencies of PARV4 and B19V DNA positive samples were compared by the chi-square test with Yates correction.

## Competing interests

The authors declare that they have no competing interests.

## Authors' contributions

FC participated in planning of the study and carried out the assays for qualitative and quantitative PARV4 and B19V DNA detection and for sequencing. KZ participated in planning of the study and helped in editing the manuscript. RF, VDG, MI, MR and SDL provided clinical samples. AA conceived and planned the study and wrote the manuscript. All authors read and approved the final manuscript.

## References

[B1] Jones MS Kapoor A Lukashov VV Simmonds P Hecht F Delwart E New DNA viruses identified in patients with acute viral infection syndrome J Virol 2005 79 8230 8236 10.1128/JVI.79.13.8230-8236.2005 15956568 PMC1143717

[B2] Lau SK Woo PC Tse H Fu CT Au WK Chen XC Tsoi HW Tsang TH Chan JS Tsang DN Li KS Tse CW Ng TK Tsang OT Zheng BJ Tam S Chan KH Zhou B Yuen KY Identification of novel porcine and bovine parvoviruses closely related to human parvovirus 4 J Gen Virol 2008 89 1840 1848 10.1099/vir.0.2008/000380-0 18632954

[B3] Fryer JF Delwart E Bernardin F Tuke PW Lukashov VV Baylis SA Analysis of two human parvovirus PARV4 genotypes identified in human plasma for fractionation J Gen Virol 2007 88 2162 2167 10.1099/vir.0.82620-0 17622618

[B4] Simmonds P Douglas J Bestetti G Longhi E Antinori S Parravicini C Corbellino M A third genotype of the human parvovirus PARV4 in sub-Saharan Africa J Gen Virol 2008 89 2299 2302 10.1099/vir.0.2008/001180-0 18753240

[B5] Sharp CP Lail A Donfield S Simmomns R Leen C Klenerman P Delwart E Gomperts ED Simmonds P High frequency of exposure to the novel human parvovirus PARV4 in hemophiliacs and injection drug users, as detected by a serological assay for PARV4 antibodies J Infect Dis 2009 200 119 125 10.1086/605646 PMC2914696 19691429

[B6] Fryer JF Kapoor A Minor PD Delwart E Baylis SA Novel parvovirus and related variant in human plasma Emerg Infect Dis 2006 12 151 154 16494735 10.3201/eid1201.050916 PMC3291395

[B7] Fryer JF Hubbard AR Baylis SA Human parvovirus PARV4 in clotting factor VIII concentrates Vox Sang 2007 93 341 347 18070279 10.1111/j.1423-0410.2007.00979.x

[B8] Fryer JF Delwart E Hecht FM Bernardin F Jones MS Shah N Baylis SA Frequent detection of the parvoviruses, PARV4 and PARV5, in plasma from blood donors and symptomatic individuals Transfusion 2007 47 1054 1061 10.1111/j.1537-2995.2007.01235.x 17524097

[B9] Lurcharchaiwong W Chieochansin T Payungporn S Theamboonlers A Poovorawan Y Parvovirus 4 (PARV4) in serum of intravenous drug users and blood donors Infection 2008 36 488 491 10.1007/s15010-008-7336-4 18759058

[B10] Simmonds P Manning A Kenneil R Carnie FW Bell JE Parenteral transmission of the novel human parvovirus PARV4 Emerg Infect Dis 2007 13 1386 1388 18252117 10.3201/eid1309.070428 PMC2857296

[B11] Manning A Russell V Eastick K Leadbetter GH Hallam N Templeton K Simmonds P Epidemiological profile and clinical associations of human bocavirus and other human parvoviruses J Infect Dis 2006 194 1283 1290 10.1086/508219 17041855 PMC7199845

[B12] Manning A Willey SJ Bell JE Simmonds P Comparison of tissue distribution, persistence, and molecular epidemiology of parvovirus B19 and novel human parvoviruses PARV 4 and human bocavirus J Infect Dis 2007 195 1345 1352 10.1086/513280 17397006 PMC7109978

[B13] Schneider B Fryer JF Reber U Fischer HP Tolba RH Baylis SA Eis-Hübinger AM Persistence of novel human parvovirus PARV4 in liver tissue of adults J Med Virol 2008 80 345 351 10.1002/jmv.21069 18098166

[B14] Botto S Bergallo M Sidoti F Terlizzi ME Astegiano S Ponti R Costa C Cavallo R Detection of PARV4, genotypes 1 and 2, in healthy and pathological clinical specimens New Microbiol 2009 32 189 192 19579698

[B15] Corcioli F Zakrzewska K Rinieri A Fanci R Innocenti M Civinini R De Giorgi V Di Lollo S Azzi A Tissue persistence of parvovirus B19 genotypes in asymptomatic persons J Med Virol 2008 80 2005 2011 10.1002/jmv.21289 18814251

[B16] Longhi E Bestetti G Acquaviva V Foschi A Piolini R Meroni L Magni C Antinori S Parravicini C Corbellino M Human parvovirus 4 in the bone marrow of Italian patients with AIDS AIDS 2007 21 1481 1483 10.1097/QAD.0b013e3281e38558 17589196

[B17] Lotze U Egerer R Glück B Zell R Sigusch H Erhardt C Heim A Kandolf R Bock T Wutzler P Figulla HR Low level myocardial parvovirus B19 persistence is a frequent finding in patients with heart disease but unrelated to ongoing myocardial injury J Med Virol 2010 82 1449 1457 10.1002/jmv.21821 20572082

[B18] Mogensen TH Jensen JMB Hamilton-Dutoit S Larsen CS Chronic hepatitis caused by persistent parvovirus B19 infection BMC Infect Dis 2010 10 246 10.1186/1471-2334-10-246 20727151 PMC2936411

[B19] Wang C Heim A Schlaphoff V Suneetha PV Stegmann KA Jiang H Krueger M Fytili P Schulz T Cornberg M Kandolf R Manns MP Bock CT Wedemeyer H Intrahepatic long-term persistence of parvovirus B19 and its role in chronic viral hepatitis J Med Virol 2009 81 2079 2088 10.1002/jmv.21638 19856479

[B20] Kühl U Pauschinger M Bock T Klingel K Schwimmbeck CP Seeberg B Krautwurm L Poller W Schultheiss HP Kandolf R Parvovirus B19 infection mimicking acute myocardial infarction Circulation 2003 108 945 950 10.1161/01.CIR.0000085168.02782.2C 12925460

[B21] Liu SC Tsai CT Wu CK Yu MF Wu MZ Lin LI Wang SS Hwang JJ Tseng YZ Chiang FT Tseng CD Human parvovirus B19 infection in patients with coronary atherosclerosis Arch Med Res 2009 40 612 617 10.1016/j.arcmed.2009.09.002 20082878

